# A cone-beam computed tomography-based morphometric comparison of mandibular molars between Han Chinese and Malays

**DOI:** 10.1016/j.jds.2025.08.026

**Published:** 2025-08-29

**Authors:** Jacob John, Wei Cheong Ngeow, Ting-Chun Shen, Lih-Jyh Fuh, Phrabhakaran Nambiar, Yen-Wen Shen, Jui-Ting Hsu

**Affiliations:** a Department of Restorative Dentistry, Faculty of Dentistry, Universiti of Malaya, Kuala Lumpur, Malaysia; b Department of Oral and Maxillofacial Clinical Sciences, Faculty of Dentistry, Universiti of Malaya, Kuala Lumpur, Malaysia; c Master Program for Biomedical Engineering, China Medical University, Taichung, Taiwan; d School of Dentistry, China Medical University, Taichung, Taiwan; e Department of Dentistry, China Medical University Hospital, Taichung, Taiwan; f Faculty of Dentistry, MAHSA University, Selangor, Malaysia; g Department of Biomedical Engineering, China Medical University, Taichung, Taiwan; h Office of Research and Development, Asia University, Taichung, Taiwan

**Keywords:** Root-to-crown ratio, Mandibular molar, Morphometry, Cone-beam computed tomography, Han Chinese, Malays

## Abstract

**Background/purpose:**

Variations in tooth and mandibular morphometry exist among ethnic groups and may have clinical and anthropological implications. This study compared the tooth length and root-to-crown ratio (RCR) of mandibular first (M1) and second (M2) molars in Han Chinese and Malays, and assessed the root length relative to mandibular height (Root-Mandible Ratio @ R: Mand).

**Materials and methods:**

One hundred twenty-one cone-beam computed tomography (CBCT) images were included. Relevant measurements were made using the CBCT software to obtain anatomical and clinical crown and root lengths. The measurements of the mesial and distal roots were averaged and used to calculate the RCR and R: Mand. Sixty-one CBCT scans of Malay patients were retrieved, and another 60 scans were of Han Chinese ethnicity.

**Results:**

There was a statistical difference in the tooth morphometry and their RCRs, with the Malays’ findings being significantly higher than the Han Chinese; the exception being the clinical-RCR (c-RCR) of M2 and the R: Mand at M2. Most parameters were generally significantly larger at M1 than at M2. The mandible height at M1 was similar in both ethnic groups, but the R: Mand of M2 was significantly higher (50.69 %) in the Han Chinese because of their low mandibular height. The Han Chinese have shorter crowns and roots for M1 and M2, and mandible height compared to the Malays at M2.

**Conclusion:**

The observed morphometric differences may reflect underlying genetic and/or environmental factors and possible clinical impacts on facial morphology and occlusion between the two ethnic groups.

## Introduction

**Figure 1:**
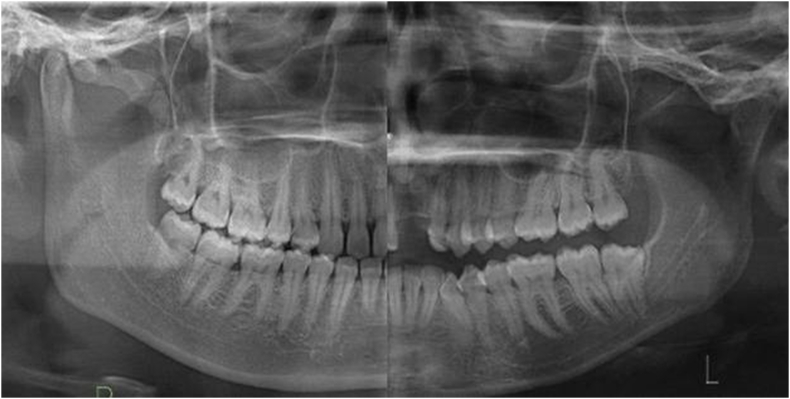
A combination of radiographic image showing two different presentations of root to mandible ratio. Left image shows a ratio of about 50 % while a higher ratio is shown in the right image.

It is acknowledged that there are genetic and race-related variations in the morphology of the mandibular first (M1) and second (M2) molars, although their root and canal anatomy have recurring features.^1^, ^2^, ^3^, ^4^ Knowledge about morphology of the mandibular molar teeth is important for anthropological and forensic analyses, especially in understanding the evolution of different ethnic groups of a same biological taxon within East and Southeast Asian populations. Their morphometric parameters are important clinically in the field of endodontics, periodontology, prosthodontics, and orthodontics, besides dentoalveolar surgery.^5^ Understanding the root lengths encased within the mandible is important to determine the risk of weakening or fracturing the mandible during exodontia. Although fractures associated with exodontia are uncommon, incidence ranging from 0.0034 to 0.0075 %^6^ has been reported. Fracture resulting from the removal of M1 and M2 accounted for 14 % of exodontia-related fractures of the mandible.^6^ Several contributing factors include the force exerted and the weakening of the mandible due to disorders such as osteoporosis. In clinical practice, clinicians occasionally observe patients with a high root length relative to the mandibular height (Fig. 1). It is suspected that this clinical feature may predispose the mandible to bone weakening, where fracture can occur due to excessive force or bone removal being implemented during exodontia.^7^

Most of our understanding of tooth lengths is derived from the Caucasians' data, last published almost 4 decades ago. On average, the length of M1 and M2 is 21.0 mm and 19.8 mm, respectively.^8^ In comparison, literature on Asians was mostly limited to Korean and Bangladeshi populations.^9^, ^10^, ^11^ In normal circumstances, the root length of a tooth is generally longer than its crown height. Their relative lengths are reported as the root-to-crown ratio (RCR), which can be an anatomical or a clinical ratio. The anatomical RCR (a-RCR) is obtained by using the cemento enamel junction (CEJ) as a reference point, while the clinical RCR (c-RCR) is obtained by using a reference line drawn from the mesial to the distal crestal bone level^12^ to distinguish the crown from the roots.

The a-RCR was used by Hölttä^13^ to determine abnormality in root development. In comparison, the c-RCR reflects the alveolar bone support that exists around teeth.^12^^,^^14^ A normal c-RCR indicates adequate support for teeth to function under acceptable physiologic stress. For this purpose, a ratio of 2.0 is deemed to be ideal, with a ratio of 1.5 being acceptable. In comparison, a ratio of 1.0 is deemed the minimal acceptable ratio.^14^ The c-RCR is used to predict the prognosis of teeth undergoing prosthodontic and orthodontic treatment.^15^ Unfavourable RCR, caused by short dental roots, may affect the long-term retention of teeth.^16^ It may complicate treatment planning, for example, in orthodontics or prosthodontics when considering anchorage or estimating the ability of a tooth to carry masticatory forces. To the best of our knowledge, very few studies have been done on the RCR of Asian populations. However, one reported study has been conducted on the Malaysian subjects^17^ while another one was performed on the Iranian population.^18^

All morphometry parameters described above can be investigated using images of cone-beam computed tomography (CBCT), which negates the need to measure extracted teeth or perform cadaveric study to measure the size of the mandible.^19^ It was the aim of this study to undertake a comparative CBCT study to determine the morphometry of M1 and M2 molars among Malays and Han Chinese. This study also tried to determine the respective a-RCR, c-RCR, and relative root length to the mandibular height (Root-Mandible ratio @ R: Mand%), to see if there is an anthropological difference between the Malays and Han Chinese. Despite the availability of some data on Korean and Bangladeshi populations, there remains a significant gap in the literature regarding detailed CBCT-based morphometric analysis of other Asian populations. By investigating multiple morphometric indices across two distinct Asian ethnic groups, this study aims to address these limitations and contribute more comprehensive anatomical data to the underrepresented Asian dental populations.

## Materials and methods

### Data source

This research received the relevant Institutional Board of Study approval from both institutions: DF OS 1703/0016 [U] (Universiti Malaya, UM) and No. CMUH 108-REC2-083 (China Medical University, CMU). One hundred and twenty-nine CBCT scans of patients taken between the years 2015 and 2016 were obtained from the Oral & Maxillofacial Radiology Unit of the Faculty of Dentistry, UM, and 232 CBCT images of patients taken between the years 2018 and 2019 were obtained from the CMU Hospital. All patients consented to contributing their imaging data for research purposes. All images were taken following a standard protocol for patient positioning.

The images obtained from the UM were captured using the i-CAT imaging system (Imaging Sciences International, Hatfield, USA). The exposure parameter (120 kVp, 3–7 mA, 20 s) and the image acquisition at 0.3 mm voxel size were done by the same radiographer. The images were obtained from scans acquired with 16 cm (diameter) and 13 cm (height) dimensions and were reconstructed using proprietary i-CAT image reconstruction software. The images obtained from the CMU were captured using Promax 3D Max (Planmeca, Helsinki, Finland). The exposure parameter (96 kV, 12.5 mA, 12 s) and the image acquisition at 0.2 mm voxel size were done by the same radiographer.

The selection criteria for study subjects were as follows:

1. Malay or Han Chinese adult individuals, regardless of gender
2. Presence of fully erupted and intact mandibular teeth with or without the third molars.
3. Presence of M1 and M2 with fully formed apices.
4. M1 and M2 with large caries, restorations, root canal treatment, or those with defect and/or associated periapical radiolucency or radiological artefact arising from metal restorative material were excluded because of possible altered coronal size.
5. Images must be free from any radiolucent or radiopaque lesion in the mandible. There should be no evidence of jaw fracture around the mandibular molar region.
6. Images with supernumeraries and unerupted teeth were excluded because the impacted or unerupted teeth might displace the molars from their original locations.
7. Images with missing upper molars were excluded because of the possibility of over-eruption of the lower molars.

**Figure 2:**
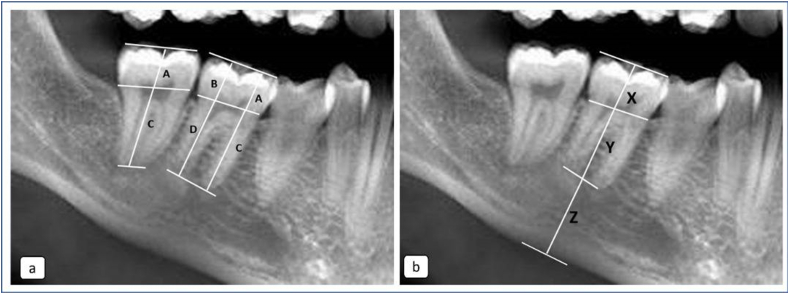
(a): The landmarks used for obtaining anatomical measurement of crown and root lengths of teeth with single and double roots. (b): The landmarks used for obtaining clinical measurements of crown and root lengths of teeth, and the height of the mandible. Note: A as the mesial crown height of the tooth; C as the mesial root length of the tooth; B as the distal crown height of the tooth; D as the distal root length of the tooth; X is the crown height of the tooth; Y is the root length of the tooth, as supported within the alveolar bone; Z is the distance from the apex of root to the bottom of the mandibular bone.

The CBCT images were analysed using a 3D software as indicated for the respective CBCT systems. All images were reworked according to axial, sagittal, and coronal planes. Using the panoramic window, the anatomical and clinical crown and root lengths, as well as mandibular height, were measured based on the methodology illustrated in Fig. 2. For performing anatomical measurements, the CEJ was used as the reference landmark separating the crown from its root(s). In M1 and M2 with 2 roots, morphometric (anatomic) measurements were obtained at both mesial and distal roots. Measurements of molars with fused roots are recorded as a single mesial root.

The a-RCR was derived from the anatomical crown height and root length obtained using the landmarks shown in Fig. 2 (a). These landmarks were identified: A is the mesial crown height of the tooth, B is the distal crown height of the tooth, C is the mesial root length of the tooth, and D is the distal root length of the tooth.

The formula to calculate a-RCR was [A/C + B/D]/2 and [A/C]/2 for multirooted and single rooted molars respectively. Based on the criteria developed by Hölttä,^13^ the a-RCR of teeth was classified into four abnormality ratios to determine if they were subjected to disturbance in root development. They were >1.6 for no disturbance, 1.2 to 1.6 for mild, 0.9 to 1.1 for moderate to severe, and <0.9 for very severe disturbance or arrested root development.

Fig. 2(b) shows three landmarks that were used to obtain the c-RCR and R: Mand%. The alveolar bone level was used as the landmark separating the clinical crown from the root that was encased within the alveolar bone. X is the crown height of the tooth, Y is the root length of the tooth, as supported within the alveolar bone, Z is the distance from the apex of the root to the bottom of the mandibular bone, and [Y + Z] is the mandible height. The formula to obtain the c-RCR was Y/X. The R: Mand% was calculated using the formula: Y/[Y + Z] x 100.

### Data analysis

The patients’ demographic data and the assigned measurement scores were recorded into Microsoft Excel 2013 software (Microsoft, Redmond, WA, USA). The mean and standard deviation, as well as 95 % confidence interval values, were calculated and compared using the SPSS Statistics 24.0 for Windows software (IBM, Armonk, NY, USA). Independent *t*-tests were used to determine the effect of gender, ethnicity, and location on the morphometry of the molars. Differences in morphometric measurements were considered statistically significant at the 5 % probability level (*P* < 0.05).

## Results

This study included 61 CBCT scans of Malay and 60 Han Chinese patients who fulfilled the selection criteria set for the study. Their socio-demographic data is shown in Table 1.

**Table 1:** Sociodemographic data of patients, including the number of mandibular molars and roots analyzed.

	Ethnicity	
	Malay	Han Chinese
Gender		
Male	35 (58.3 %)	29 (48.3 %)
Female	26 (41.7 %)	31 (51.7 %)
Age: Mean (SD)	29.8 (9.6) years	34.1 (12.6) years
Number of teeth measured	226	225
Number of roots measured	465	450

SD = standard deviation.

### Morphometric measurements of molars

The distribution of morphometric measurements obtained for the anatomical and clinical crown heights and root lengths of M1 and M2, together with their corresponding a-RCR and c-RCR in both ethnic groups, is shown in Table 2.

**Table 2:** The *anatomical* and *clinical* crown heights and root lengths of mandibular first and second molars.

			Malays∗				Han Chinese∗			
			*Anatomical*		*Clinical*		*Anatomical*		*Clinical*	
			Mean (SD)	Average	Mean (SD)	Average	Mean (SD)	Average	Mean (SD)	Average
**M1**	**Crown∗∗**	**Right**	6.69 (0.84)	6.75 (0.86)	7.50 (0.79)	7.48 (0.88)	6.36 (0.66)	6.39 (0.61)	7.29 (0.97)	7.28 (0.88)
		**Left**	6.80 (0.86)		7.46 (0.96)		6.42 (0.55)		7.27 (0.76)	
	**Root∗∗**	**Right**	12.32 (1.49)^ϒ^	12.69 (1.77)	11.51 (1.40)	11.73 (1.53)	11.47 (1.76)	11.49 (1.71)	10.40 (1.94)	10.60 (1.89)
		**Left**	13.07 (1.93)^ϒ^		11.96 (1.53)		11.51 (1.65)		10.82 (1.80)	
	**RCR**	**Right**	1.87 (0.32)	1.91 (0.38)	1.55 (0.25)	1.59 (0.29)	1.80 (0.34)	1.80 (0.31)	1.44 (0.32)	1.47 (0.31)
		**Left**	1.96 (0.43)		1.63 (0.33)		1.79 (0.26)		1.49 (0.30)	
**M2**	**Crown**	**Right**	6.24 (0.78)^ϒ^	6.41 (0.81)	7.00 (1.06)	7.08 (1.60)	6.65 (0.67)	6.63 (0.74)	6.75 (1.13)	6.84 (1.18)
		**Left**	6.56 (0.80) ^ϒ^		7.17 (0.96)		6.61 (0.82)		6.92 (1.22)	
	**Root**	**Right**	11.61 (0.85)	11.73 (1.87)	11.18 (1.88)	11.04 (1.99)	11.15 (1.82)	11.09 (1.85)	11.03 (2.04)	10.80 (2.02)
		**Left**	11.84 (1.85)		10.91 (2.06)		11.1 (1.87)		10.55 (1.96)	
	**RCR**	**Right**	1.89 (0.40)	1.87 (0.41)	1.63 (0.037)	1.60 (0.40)	1.70 (0.27)	1.71 (0.31)	1.72 (0.52)	1.66 (0.51)
		**Left**	1.84 (0.42)		1.56 (0.42)		1.71 (0.35)		1.60 (0.49)	

M1 = first molar; M2 = second molar; SD = standard deviation; RCR = root-to-crown ratio.

For *Anatomical* M1∗Independent t-test, comparing ethnic groups, *P* < 0.05; ∗∗Independent t-test, comparing right and left crown and root lengths, *P* > 0.05; ^ϒ^ Independent t-test; *P* = 0.024.

For *Anatomical* M2 ∗Independent t-test, comparing ethnic groups *P* < 0.05, except for crown height of M2 (*P* = 0.468); ∗∗Independent t-test, comparing right and left crown and root lengths *P* > 0.05; ^ϒ^ Independent t-test; *P* = 0.038.

For *Clinical* M1 ∗Independent t-test, comparing ethnic groups *P* > 0.05, except for root length (*P* < 0.001) and RCR (*P* = 0.003); ∗∗Independent t-test, comparing right and left crown and root lengths *P* > 0.05.

For *Clinical* M2 ∗∗Independent t-test, comparing ethnic groups *P* > 0.05; ∗∗Independent t-test, comparing right and left crown and root lengths *P* > 0.05.

Except for the anatomical root length of M1 and anatomical crown length of M2 of the Malays, statistical analyses showed no significant difference between mesial and distal crown heights and root lengths, and between measurements obtained from the right and left sides for both ethnic groups (independent t-test; *P* > 0.05). There was also no gender related difference in the measurements among the Malays (independent t-test; *P* > 0.05). Data on the Han Chinese, however, showed a significant difference relating to M1. The anatomical crown (independent t-test; *P* = 0.0090) and root (independent t-test; *P* = 0.0026) were significantly larger in Han Chinese males.

By pooling the data for both the mesial and distal roots, and left and right sides, the average anatomical crown height of M1 was 6.75 (0.86) mm, while that of M2 was 6.41 (0.81) mm in the Malays (independent t-test; *P* = 0.03). In comparison, the average crown height of M1 was significantly shorter at 6.39 (0.61) mm in the Han Chinese. However, the average crown height of M2 was only slightly longer at 6.63 (0.74) mm in the Han Chinese. The root length of M1 in the Malays was significantly longer at 12.69 (1.77) mm in contrast to the 11.73 (1.87) mm observed for M2 (independent t-test; *P* < 0.0001). In comparison, M1 and M2 had significantly shorter root lengths, 11.49 (1.7) mm and 11.09 (1.85) mm, among the Han Chinese. By adding the crown heights and root lengths of each M1 and M2, recalculation showed that the tooth lengths for M1 and M2 were 19.41 (1.54) mm and 18.15 (1.77) mm, respectively, in the Malays. The anatomical tooth lengths for M1 and M2 of the Han Chinese were comparable at 17.87 (1.97) mm and 17.81 (2.08) mm, respectively.

Similarly, by pooling the data for both the mesial and distal roots, and left and right sides, the average clinical crown height of M1 was 7.48 (0.88) mm, while that of M2 was 7.08 (1.60) mm in the Malays (independent t-test; *P* = 0.0015). In comparison, the average crown height of M1 was significantly shorter at 7.28 (0.88) mm in the Han Chinese. However, the average crown height of M2 was only slightly shorter at 6.84 (1.18) mm in the Han Chinese. The root length of M1 in the Malays was significantly longer at 11.73 (1.53) mm in contrast to the 11.04 (1.99) mm observed for M2 (independent t-test; *P* = 0.004). In comparison, M1 and M2 had significantly shorter root lengths, 10.6 (1.89) mm and 11.08 (2.02) mm, among the Han Chinese. By adding the crown heights and root lengths of each M1 and M2, recalculation showed that the tooth lengths for M1 and M2 were 19.20 (1.63) mm and 18.12 (2.00) mm, respectively, in the Malays. The clinical tooth lengths for M1 and M2 of the Han Chinese were comparable at 17.82 (2.00) mm and 17.68 (2.15) mm, respectively.

### Root-to-crown ratio

**Figure 3:**
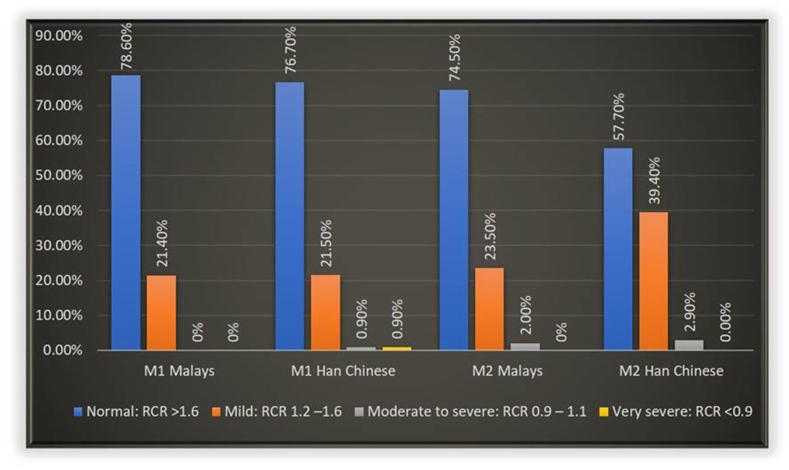
The distribution of *a-*RCR according to different types of root development disturbances. Note: M1 = first molar; M2 = second molar; RCR = root-to-crown ratio.

The average a-RCR for M1 and M2 was 1.91 (0.38) and 1.87 (0.41), respectively, for the Malays, showing no statistically significant difference (independent t-test; *P* = 0.364). In comparison, the corresponding a-RCR for the Han Chinese were significantly lower at 1.80 (0.31) and 1.71 (0.31), respectively. There was a statistically significant difference between a-RCR at M1 and M2 in Han Chinese (Independent t-test; *P* = 0.029). When the a-RCRs were further categorised according to the abnormality ratio developed by Hölttä et al., (2002), the findings suggests that between 21.4 % (M1) and 23.5 % (M2) of the molars in the Malays and 23.3 % of M1 and 42.3 % of M2 in Han Chinese suffered from some form of disturbances in the root development (Fig. 3). In the Malay subjects, 2.0 % suffered from moderate to severe disturbance. A significantly large percentage of M2 of the Han Chinese had ratios indicative of mild root disturbance (39.40 %) and moderate to severe disturbance (2.90 %).

The clinical crown heights, root lengths, and the c-RCR measurements for mandibular M1 and M2 of both ethnic groups are shown in Table 2. Because measurements were done at the alveolar bone level, the clinical crown height increased, while the clinical root lengths reduced, when compared to their anatomical counterparts. There was no difference in the crown height and root length between the right and left sides (independent t-test; *P* > 0.05). The c-RCR for the Malays were 1.59 (0.29) for M1 and 1.60 (0.40) for M2, respectively, showing no statistically significant difference (independent t-test; *P* = 0.877), as observed in their corresponding a-RCR. In comparison, the c-RCR for the Han Chinese was significantly lower at 1.47 (0.31) for M1 but significantly higher at 1.66 (0.51) for M2. Similar to the observation of the a-RCR measurement, there was a statistically significant difference between the c-RCR of M1 and M2 (independent t-test; *P* < 0.001).

### The root-mandible ratio

Table 3 shows the clinical root length and mandibular heights at M1 and M2. In both the ethnic groups, the right and left mandible heights at M1 and M2 were not significantly different. Hence, the data were pooled to arrive at the overall mandible height of 26.21 (5.83) mm [95%CI] at M1 and 24.78 (3.52) [95 % CI] mm at M2. For the mandible height at M1, there was no significant difference between the Malays and Han Chinese, with the Han Chinese presenting with 26.24 (3.80) mm being the mandibular height. However, for the mandible height at M2, the mandible height was shorter in Han Chinese, measuring at only 22.05 (4.46) mm. The mandible heights were significantly higher at M1 than at M2 in both ethnic groups.

**Table 3:** The *clinical* root length and mandibular heights of the mandibular first and second molars.

	Malays^a^			Han Chinese^a^		
	Root length	Mandible height	R: Mand %	Root length	Mandible height	R: Mand %
M1						
**Right**^b^	11.51 (1.40) mm	27.03 (3.15) mm	42.66 (9.26)	10.40 (1.94) mm	26.39 (3.75) mm	39.80 (7.32)
**Left**^b^	11.95 (1.612) mm	27.79 (3.75) mm	43.48 (5.54)	10.82 (1.80) mm	26.08 (3.84) mm	42.40 (9.62)
**Overall mean**	11.73 (1.53) mm	26.21 (5.83) mm	43.06 (7.64)	10.60 (1.89) mm	26.24 (3.80) mm	41.05 (8.60)
**M2**						
**Right**^b^	11.18 (1.88) mm	24.42 (3.36) mm	46.33 (7.91)	11.03 (2.04) mm	22.10 (4.25) mm	51.64 (12.75)
**Left**^b^	10.91 (2.06) mm	25.13 (3.65) mm	44.12 (8.57)	10.55 (1.96) mm	22.01 (4.66) mm	49.65 (11.97)
**Overall mean**	11.04 (1.99) mm	24.78 (3.52) mm	45.20 (8.29)	10.80 (2.02) mm	22.05 (4.46) mm	50.67 (12.42)

M1 = first molar; M2 = second molar; R: Mand % = root-mandible ratio.

a Independent t-test *P* < 0.05 when comparing ethnic groups, except for root-mandible ratio of M1 (*P* = 0.064).

b Independent t-test *P* > 0.05 when comparing right and left root-mandible ratio.

The R: Mand% used to determine the relative length of roots embedded in the mandible. In the Malays, the root-mandible ratios at these for 4 sites were 42.66 % (right M1), 43.48 % (left M1), 46.33 % (right M2) and 44.12 % (left M2). These differences were only statistically significant between M1 (43.06 %; 95%CI) and M2 (45.20 %; 95%CI) (Independent t-test; *P* = 0.047). In the Han Chinese, the Root-Mandible Ratios at the 4 sites were 39.8 % (right M1), 42.4 % (left M1), 51.7 % (right M2), and 49.7 % (left M2). These differences were also statistically significant between M1 (41.05 %; 95%CI) and M2 (50.69 %; 95%CI) (Independent t-test; *P* < 0.001). The former ratio was lower than those observed in the Malays, while the latter ratio was significantly higher than those observed in the Malays.

## Discussion

East and Southeast Asian populations have been reported to have two types of teeth, namely the Sinodont-type and the Sundadont-type. Because of this, Ishii^20^ pointed out that populations in Northeastern Asia has different dental trait compared to those residing in Southeast Asia. In their case, a lower prevalence of radix endomolaris was observed in the latter. This difference may be related to the difference in East Asian populations, with the Sinodont type of teeth being common among northeastern Asians and the Sundadont type being common among southeastern Asians.^21^ Malaysia, Singapore, and Thailand are situated in Southeast Asia, and their populations are deemed to have Sundadont-type teeth. In contrast, Taiwan is located at the edge of Southeast Asia, but its population originates mainly from the Northeastern part of Asia. Hence, they are more likely to have Sinodont-type teeth. The morphometric findings of this current study attest to this possible genetic and/or environmental factors. There were statistical differences in the tooth morphometry and their RCRs, mandible height, and Root-Mandible Ratio, with the Malays’ findings being significantly higher than the Han Chinese; the exception being the c-RCR of M2 and the Root-Mandible ratio at M2.

A comparison of the findings of our tooth lengths against two reports from the West is shown in Table 4. As can be seen, the tooth lengths in these patients are shorter than that reported by Black^22^ and Bjorndal^23^ for the Caucasians. Alam reported that the average length of M1 was 20.28 mm in Bangladeshi, which they deemed as being shorter than their Caucasoid counterpart. The finding that the teeth/roots in our findings among Han Chinese and Malays are consistent with previous studies on Asian populations. Yaacob^2^ reported that the anatomical roots of Mongoloids are shorter, but the root trunks are better developed. In comparison, the tooth length of M1 in the present study was shorter than the 20.28 mm length reported by Alam.^9^

**Table 4:** Comparison of tooth measurements from the present study with two reported references from Western literature.

		Mean length (SD)[Min – Max] in mm			
		Black (1902)	Bjorndal et al. (1974)	Current study (Malay)	Current study (Han Chinese)
M1	Tooth length	21.0 [18.0–24.0]	22.0 (1.4) [19.3–25.0]	19.41 (1.54)	17.87 (1.97) [13.05–22.80]
	Crown height	7.7 [7.0–10.0]	8.3 (0.7) [6.4–10.2]	6.75 (0.86) [5.04–8.80]	6.39 (0.61)
	Mesial root length	13.2 [11.0–15.0]	15.1 (1.2) [11.9–17.3]	12.69 (1.77) [9.12–22.93] (combined)	11.49 (1.71) [7.85–16.05] (combined)
	Distal root length				
M2	Tooth length	19.8 [18.0–22.0]	21.7 (1.5) [19.0–25.8]	18.15 (1.77)	17.81 (2.08) [13.57–22.76]
	Crown height	6.9 [6.0–8.0]	8.7 (0.9) [6.8–13.1]	6.41 (0.81) [4.37–8.66]	6.63 (0.74) [4.69–9.08]
	Mesial root length	12.9 [12.0–14.0]	13.8 (1.3) [10.3–17.6]	11.73 (1.86) [5.89–15.60] (combined)	11.09 (1.84) [7.24–15.80] (combined)
	Distal root length		13.4 (1.3) [10.3–17.0]		

M1 = first molar; M2 = second molar; SD = standard deviation; Min = Minimum; Max = Maximum.

The M1 and M2 root length of Homo sapiens of Caucasoid origin have been reported to be 14.17 ± 1.16 mm and 14.06 ± 1.63 mm respectively.^24^ The M1 root length of the Malays (12.69 mm) and Han Chinese (11.49 mm) in the present study is close to the mesial and distal root lengths of 12.19 (1.13) mm and 11.53 (1.32) mm reported for the Chinese subjects.^25^ This finding suggests that dental treatments such as root canal therapy and post placement in fixed prosthodontics can be undertaken using standard instruments.

Studies among other East and Southeast populations, such as the Korean study^12^ which used the root length as measured of those within the alveolar bone, found that the clinical RCR of M1 as 1.64 (0.19) and M2 as 1.47 (0.23). The mean clinical RCR for M1 of both our ethnic groups was lower than the ratios reported among the Koreans. However, the clinical RCR of M2 of Malays and Han Chinese is higher than that reported by the Koreans.^12^ The findings on M2 with regard to its shorter root, high c-RCR, and the higher percentage of Root-Mandible ratio in relation to its significantly reduced mandibular height might be due to the different growth pattern and ethnic differences. Precautions should be addressed for surgical consideration in the posterior mandibular region.

A recent study^26^ suggested that teeth morphometry has forensic implications due to sexual dimorphism of the mandibular first molar. The authors found that the cervico-incisal (crown) height of the first molar was a reliable predictor for gender, with a high accuracy in predicting males. This finding can be tested on the current two studied groups in the future as the previous study was done on a Caucasoid sample.

This study confirms that Han Chinese individuals exhibit shorter molar crown and root lengths, along with reduced mandibular height in the second molar region, which aligns with CBCT-based observations in other East Asian cohorts.^27^, ^28^, ^29^ These anatomical variations substantially affect clinical decision-making across multiple dental disciplines:

1. Endodontics: Shorter root lengths in mandibular molars limit working length and heighten the risk of over-instrumentation or perforation. Although this study did not directly assess canal morphology, Han Chinese CBCT data show a prevalence of 44.7 % for C-shaped canals in mandibular second molars among a local cohort, emphasizing the relevance of anatomical complexity in endodontic planning.^30^ Consequently, clinicians should adopt conservative instrumentation protocols and utilize preoperative CBCT imaging when available to identify complex configurations and avoid procedural errors.
2. Prosthodontics and Periodontics: Reduced root length diminishes periodontal support and increases vulnerability to occlusal trauma. This necessitates occlusal adjustment and possible splinting, particularly in patients with parafunctional habits.^31^
3. Implantology and Surgery: Shorter roots and lower mandibular height restrict available bone volume, potentially requiring shorter implants or bone augmentation. Implant planning must carefully consider inferior alveolar nerve.

Overall, population-specific anatomical characteristics must be incorporated into individualized treatment planning to enhance safety and success rates.

This study is limited by its retrospective design and reliance on CBCT scans obtained for clinical rather than research purposes, which may introduce sampling bias. Additionally, the study population was limited to Malay and Han Chinese, and the findings may not be generalizable to other Asian populations. The lack of genetic data also limits the ability to link morphometric variations to specific genetic factors directly. Future studies could expand the sample size, include other East and Southeast Asian ethnic groups, and incorporate genomic analysis to explore potential genetic determinants of dental morphology. Longitudinal or functional studies may also help clarify the clinical relevance of RCR and root-to-mandible ratios in dental procedures such as extraction, implant planning, or prosthodontics.

In conclusion, the Han Chinese population exhibits distinct molar and mandibular anatomical traits—shorter crown and root lengths and reduced mandibular height. These factors significantly influence clinical outcomes in endodontics, prosthodontics, periodontics, and implantology. Integrating CBCT imaging, customized occlusal schemes, and tailored restorative or surgical strategies is essential to optimize dental treatment outcomes in this group.

## Declaration of competing interest

The authors have no conflicts of interest relevant to this article.
